# Emotional and Behavioral Problems Among Siblings of Children With Attention-Deficit/Hyperactivity Disorder in a Tertiary Child Psychiatry Setting in Bangladesh: A Cross-Sectional Study

**DOI:** 10.7759/cureus.115465

**Published:** 2026-08-30

**Authors:** A.H.M Mostofa Kamal, Md. Bahar Hossain, Md Al‑Imran Mim, Ananya Kar, Iffat T Prianka, Sumaiya B Jalil, Shelina Shahid, Nahid M Morshed

**Affiliations:** 1 Psychiatry, Monirampur Upazila Health Complex, Jashore, BGD; 2 Psychiatry, Central Police Hospital, Dhaka, BGD; 3 Psychiatry, Dinajpur Medical College Hospital, Dinajpur, BGD; 4 Psychiatry, Bangabandhu Sheikh Mujib Medical University, Dhaka, BGD; 5 Community Medicine and Public Health, Ibrahim Medical College, Dhaka, BGD; 6 Psychiatry, National Institute of Mental Health, Dhaka, BGD; 7 Psychiatry, Bangladesh Medical University, Dhaka, BGD; 8 Child and Adolescent Psychiatry, Bangladesh Medical University, Dhaka, BGD

**Keywords:** adhd, bangladesh, child and adolescent mental health, emotional and behavioral problems, sdq, siblings

## Abstract

Background: Siblings of children with attention-deficit/hyperactivity disorder (ADHD) may experience emotional and behavioral difficulties. Shared genetic and environmental factors, family stress, caregiving demands, and potentially unequal parental attention may contribute to these difficulties. However, the mental health needs of these siblings may be overlooked.

Methods: This cross-sectional descriptive study included 95 siblings aged 4-17 years of children with ADHD attending the Department of Psychiatry, BSMMU. Sociodemographic information was collected using a semi-structured questionnaire. Emotional and behavioral difficulties were assessed using the Bangla parent-report Strengths and Difficulties Questionnaire (SDQ); a self-report SDQ was additionally collected from siblings aged 11-17 years. Categorical associations were examined using chi-square tests, and findings involving small subgroup frequencies were interpreted cautiously.

Results: The mean age of siblings was 7.21 ± 3.01 years; 72.6% were male and 77.9% lived in nuclear families. Abnormal SDQ scores were identified for emotional problems in three siblings (3.2%), conduct problems in 11 siblings (11.6%), hyperactivity problems in seven siblings (7.4%), peer problems in 12 siblings (12.6%), and prosocial-behavior problems in two siblings (2.1%).

Conclusion: Abnormal SDQ scores were identified in a proportion of siblings of children with ADHD attending this tertiary-care setting. These findings suggest that clinicians may consider asking about sibling wellbeing during family assessment. Larger multicenter studies with appropriate comparison groups are needed to clarify the extent of these difficulties and to determine whether routine sibling-focused screening should be incorporated into ADHD care.

## Introduction

Attention-deficit/hyperactivity disorder (ADHD) is a neurodevelopmental disorder characterized by developmentally inappropriate levels of inattention, hyperactivity, and impulsivity [1]. It is among the most common psychiatric disorders affecting children and adolescents and is associated with substantial academic, social, and familial impairment [2,3]. A recent umbrella review of meta-analyses estimated the global prevalence of ADHD among children and adolescents at 8.0% (95% CI: 6.0%-10.0%), although estimates vary according to diagnostic criteria, study population, informant, and methodology. ADHD is also recognized among child psychiatric populations in Bangladesh [4,5].

The impact of ADHD extends beyond the diagnosed child. Parents of children with ADHD often experience greater stress, more conflictual parent-child interactions, and higher caregiving burden than parents of unaffected children [6,7]. These family-level pressures may influence sibling relationships, which are central to children’s emotional development, social learning, and day-to-day adjustment [8,9].

Siblings of children with ADHD may be particularly vulnerable to emotional and behavioral difficulties. Previous studies have described increased loneliness, resentment, conflict, reduced warmth, poorer quality of life, and elevated internalizing and externalizing symptoms in these siblings. At the family level, siblings may experience conflict with the child with ADHD, reduced warmth in sibling relationships, perceived unequal parental attention, disrupted routines, and additional caregiving responsibilities. Some siblings report interference with study, sleep, privacy, hobbies, and family activities, as well as feelings of resentment, loneliness, worry, envy, or neglect. These experiences may also extend beyond the home by affecting peer relationships, social participation, and confidence during play activities, school-related interactions, family outings, and community gatherings [10-15]. In addition, siblings of children with ADHD may have an increased risk of ADHD and other psychiatric disorders, reflecting shared familial vulnerability and environmental influences within the family [16,17]. A study from Thailand reported emotional and behavioral difficulties among siblings of children with ADHD, highlighting the need for further research across diverse cultural and clinical settings [18].

Despite increasing recognition of family-centered approaches in child psychiatry, sibling mental health is commonly overlooked in routine clinical practice. In Bangladesh, previous child mental health research has primarily described psychiatric disorders among children [5]. International literature on families of children with ADHD has largely focused on parenting stress, parenting practices, and the experiences of parents [6,7]. To our knowledge, no published Bangladeshi study has specifically examined emotional and behavioral problems among siblings of children with ADHD using a standardized screening instrument. Moreover, the available Bangladeshi literature has not described the distribution of Strengths and Difficulties Questionnaire (SDQ) problem domains among these siblings or explored their associations with sociodemographic and family-related factors, including parental education, parental occupation, family psychiatric history, number of siblings, and caregiving time. Therefore, the primary objective of the present study was to assess the distribution and types of parent-reported emotional and behavioral difficulties among siblings of children with ADHD attending a tertiary psychiatric service in Bangladesh. The secondary objective was to explore bivariate associations between SDQ domain scores and selected sociodemographic and family-related factors.

## Materials and methods

Study design and setting

This cross-sectional descriptive study was conducted from March 2022 to August 2024 at the Department of Psychiatry, Bangabandhu Sheikh Mujib Medical University, Dhaka, Bangladesh, including the inpatient and outpatient departments, the CAMH Clinic, and the ADHD Clinic. Participants were recruited using purposive sampling from eligible families attending these services. Parents accompanying children with ADHD diagnosed by consultant psychiatrists were approached and screened against the study eligibility criteria.

Participants and sampling

The study population comprised biological siblings aged 4-17 years of children with ADHD attending the inpatient or outpatient departments, CAMH Clinic, or ADHD Clinic at BSMMU with their biological parents. Parents of children with ADHD diagnosed by consultant psychiatrists were approached and screened for eligibility using purposive sampling. Siblings with a parent-reported prior psychiatric diagnosis made by a qualified mental-health professional or a chronic medical illness requiring ongoing care, such as asthma or congenital heart disease, were excluded.

The sample size was calculated using the protocol-specified formula:

n = [ 1.96√{π(1 - π)} + v√{π₀(1 - π₀)} ]² / (π - π₀)²

Where

π = 0.596, approximated to 0.60, is the anticipated proportion from the Bangladeshi clinic-based SDQ study.

π₀ = 0.45 is the reference proportion.

v = 1 - (π - π₀) = 1 - (0.60 - 0.45) = 0.85.

The anticipated proportion was π=0.596\pi=0.596π=0.596, approximated to 0.60, based on the reported mean prevalence of any psychiatric disorder assessed using the SDQ in clinic samples in Bangladesh [19]. The reference proportion was π0=0.45\pi_0=0.45π0​=0.45, representing an anticipated absolute difference of 0.15. The protocol parameter was calculated as v=1−(π−π0)=1−(0.60−0.45)=0.85v=1-(\pi-\pi_0)=1-(0.60-0.45)=0.85v=1−(π−π0​)=1−(0.60−0.45)=0.85.

n = [1.96 × √(0.60 × (1 - 0.60)) + 0.85 × √(0.45 × (1 - 0.45))]² / (0.60 - 0.45)²

The value was rounded up to 86; with a 10% allowance for incomplete data, the target sample size was 95 siblings.

A total of 118 families were assessed, 102 were recruited, and 95 siblings with complete data were analyzed. Families were excluded because of non-consent, ineligible sibling age, attendance with a relative other than a parent, pre-existing psychiatric disorder, chronic medical illness, or missing data. A flowchart of participant inclusion is presented in Figure 1.

**Figure 1:**
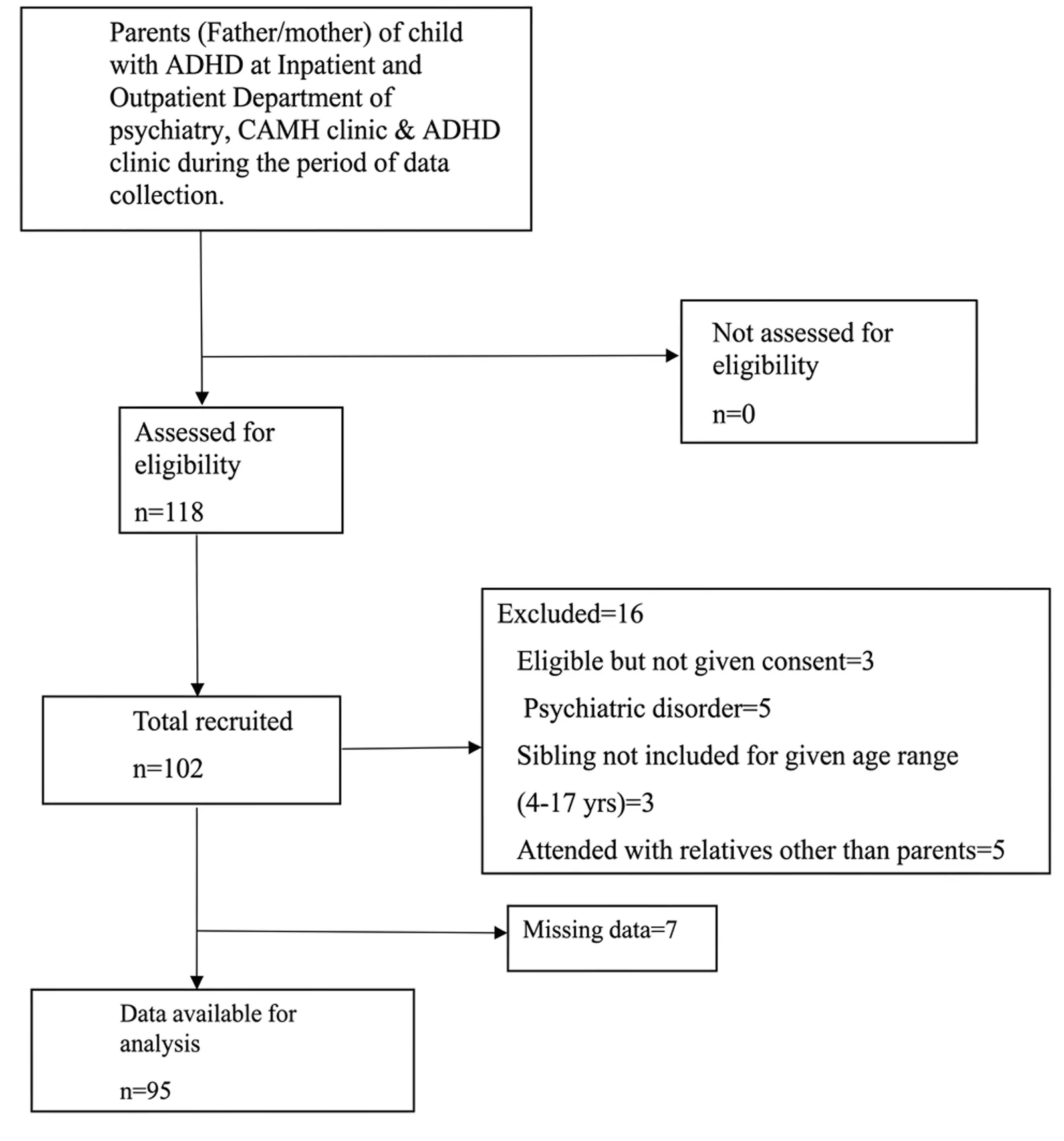
Flowchart of participant inclusion

Measures

Emotional and behavioral difficulties were assessed using the Bangla parent-report version of the Strengths and Difficulties Questionnaire (SDQ), completed for all 95 siblings aged 4-17 years. The SDQ comprises 25 items across five subscales: emotional symptoms, conduct problems, hyperactivity/inattention, peer problems, and prosocial behavior. Each subscale is scored from 0 to 10. The total difficulties score, ranging from 0 to 40, is calculated by summing the emotional symptoms, conduct problems, hyperactivity/inattention, and peer problem subscales; the prosocial-behavior subscale is not included. Scores were categorized as normal, borderline, or abnormal. For the parent-report SDQ total difficulties score, the cut-offs were normal (0-13), borderline (14-16), and abnormal (17-40). The Bengali parent-report SDQ has previously been validated in Bangladesh. For bivariate analyses, borderline and abnormal categories were combined and compared with the normal category [19,20]. The parent-report Bangla SDQ was used for all statistical analyses of the 95 participating siblings. Although self-report SDQ data were also collected from siblings aged 11-17 years, these data were not included in the reported analyses in order to maintain a consistent informant across the full study sample.

Parent-reported sibling involvement in caring for the child with ADHD was assessed using the parent-completed sociodemographic questionnaire. Parents categorized the participating sibling’s involvement as “almost always,” “occasionally,” or “no involvement.” No predefined quantitative time thresholds were used; therefore, this variable reflects parent-reported caregiving frequency rather than duration of care.

Internalizing and externalizing composite scores were calculated from the parent-report SDQ. The internalizing score was the sum of the emotional-symptoms and peer-problems subscales, whereas the externalizing score was the sum of the conduct-problems and hyperactivity/inattention subscales; both composite scores ranged from 0 to 20.

This study was approved by the Institutional Review Board of Bangabandhu Sheikh Mujib Medical University, Dhaka, Bangladesh (Approval No. BSMMU/2023/10633). All procedures involving human participants were conducted in accordance with the ethical standards of the institutional research committee and the 1964 Declaration of Helsinki and its later amendments. Written informed consent was obtained from parents or legal guardians, and assent was obtained from age-appropriate siblings. No animals were involved in this study.

Data collection procedure

After approval of the study protocol, the investigator approached eligible families at the study settings, explained the aims and procedures, obtained written informed consent, and administered the study instruments. Parents completed the sociodemographic questionnaire and the parent version of the Bangla SDQ. Siblings aged 11-17 years also completed the self-report SDQ version. Privacy and confidentiality were maintained throughout the process.

Statistical analysis

Data were edited, coded, entered, and analyzed using IBM SPSS Statistics for Windows, Version 26 (Released 2018; IBM Corp., Armonk, New York, United States). Descriptive analyses were used to summarize sociodemographic characteristics and emotional and behavioral problems. Chi-square tests were used to examine associations between explanatory variables and SDQ domains. For these analyses, borderline and abnormal SDQ categories were grouped as abnormal, while normal scores were retained as normal. Statistical significance was set at p < 0.05.

## Results

Participant flow and baseline characteristics

A total of 118 families were initially assessed for eligibility, 102 were recruited, and 95 siblings of children with ADHD were finally analyzed after excluding records with missing data. The mean age of the siblings was 7.21 ± 3.01 years. Most siblings were aged 4-9 years (80, 84.2%), were male (69, 72.6%), and were from nuclear families (74, 77.9%), and most participants, 92 (96.8%), had one sibling, meaning that they had one other child in the family; three (3.2%) had two siblings. More than four‑fifths of parents (79, 83.2%) reported that they almost always spent time taking care of the child with ADHD. Table 1 shows the sociodemographic and family profile of the study population.

**Table 1:** Sociodemographic characteristics of siblings of children with ADHD (N = 95) K: thousand; BDT: Bangladeshi Taka; ADHD: attention‑deficit/hyperactivity disorder.

Variable	Category	Frequency (n)	Percentage (%)
Age (year)	4 to 9	80	84.2
	10 to 17	15	15.8
Gender	Male	69	72.6
	Female	26	27.4
Family type	Nuclear	74	77.9
	Joint	21	22.1
Number of siblings	One	92	96.8
	Two	3	3.2
Father’s occupation	Labor	4	4.2
	Business	41	43.2
	Service	49	51.6
	Others	1	1.1
Mother’s occupation	Housewife	81	85.3
	Service	13	13.7
	Business	1	1.1
Religion	Islam	87	91.6
	Others	8	8.4
Monthly income	10,000-30,000 BDT	9	9.5
	>30,000 BDT	86	90.5
Family history of psychiatric disorder	Present	22	23.2
	Absent	73	76.8
Birth order	First	51	53.7
	Second	44	46.3
Parent-reported sibling involvement in caring for the child with ADHD	Almost always	79	83.2
	Occasionally	14	14.7
	No Involvement	2	2.1

Figure 2 shows the distribution of fathers’ and mothers’ educational attainment among the study participants’ families. Graduate-level education was the most common category for both fathers (74.7%) and mothers (47.4%), while mothers more often had primary, secondary, or higher-secondary education than fathers.

**Figure 2:**
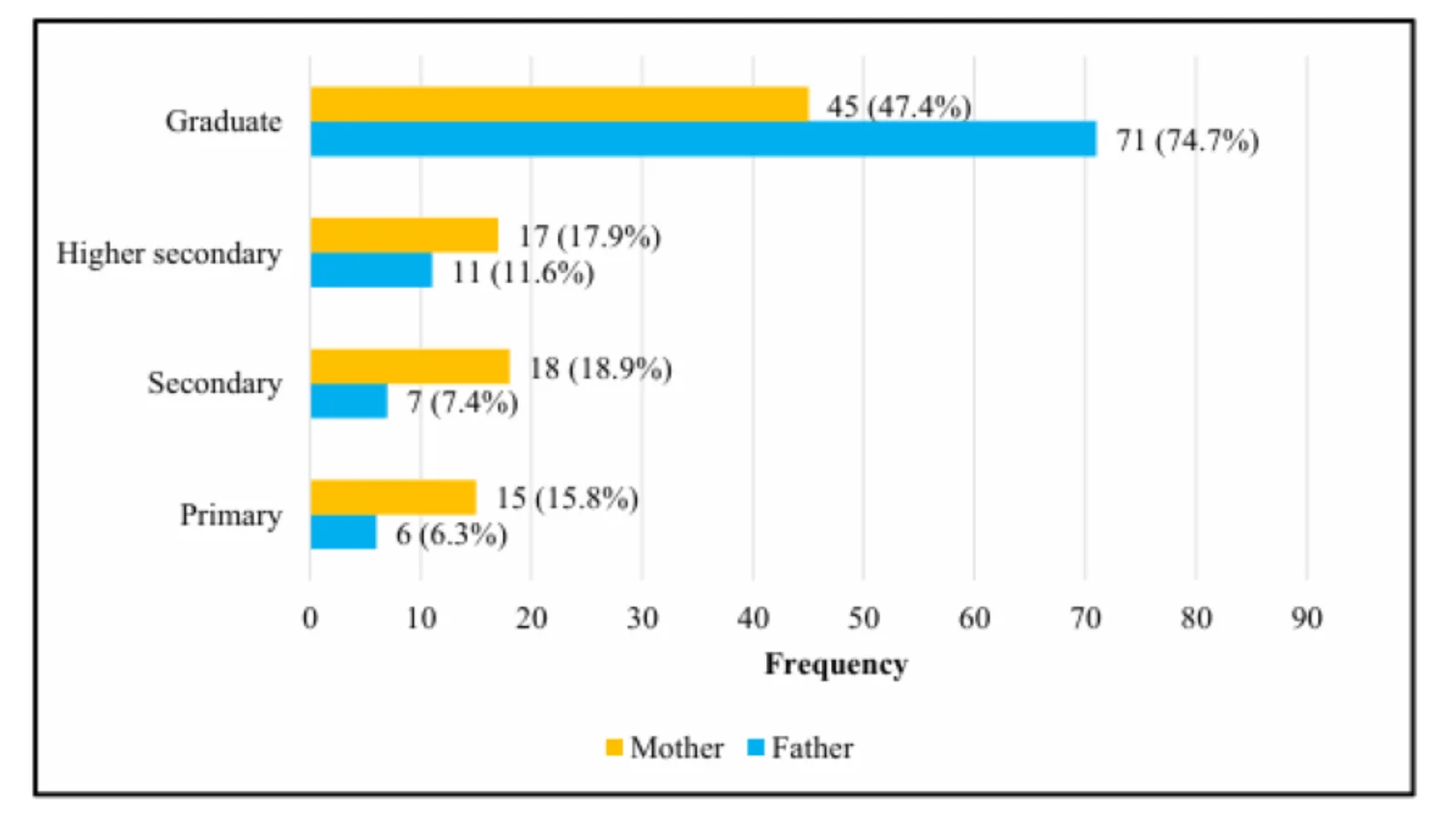
Educational attainment of parents of siblings of children with ADHD (N = 95). Educational categories were defined as primary (grades I–V), secondary (grades VI–X), higher secondary (grades XI–XII), and graduate or above (bachelor’s degree or higher).

Distribution of SDQ scores

The mean total SDQ difficulties score was 8.83 ± 4.05. Most siblings fell in the normal range for total difficulties (81, 85.3%), while seven (7.4%) were borderline and seven (7.4%) were abnormal. Across subscales, abnormal scores were found in three (3.2%) for emotional problems, 11 (11.6%) for conduct problems, seven (7.4%) for hyperactivity problems, 12 (12.6%) for peer problems, and two (2.1%) for prosocial behavior problems.

Peer problems had the highest borderline proportion (24.2%) and the highest abnormal prevalence among the relational domains, suggesting that interpersonal adjustment may be particularly affected in this group. Conduct problems were the next most frequent abnormal domain. Prosocial behavior remained normal for the large majority of participants.

Table 2 presents the exact SDQ distribution by domain.

**Table 2:** Distribution of parent-reported Strengths and Difficulties Questionnaire (SDQ) scores among siblings of children with ADHD (N = 95). *Each SDQ subscale score ranges from 0 to 10. For the prosocial-behavior subscale, lower scores indicate greater difficulty. SDQ categories indicate screening-score ranges and do not represent clinical diagnoses. ADHD: attention‑deficit/hyperactivity disorder.

Scales*	Mean ± SD score	Normal (n/%)	Borderline (n/%)	Abnormal (n/%)
Total score (range 0-40)	8.83 ± 4.05	81 (85.3)	7 (7.4)	7 (7.4)
Emotional problems	2.13 ± 1.10	87 (91.6)	5 (5.3)	3 (3.2)
Conduct problems	1.39 ± 1.70	78 (82.1)	6 (6.3)	11 (11.6)
Hyperactivity problems	2.82 ± 1.95	84 (88.4)	4 (4.2)	7 (7.4)
Peer problems	2.49 ± 0.93	60 (63.2)	23 (24.2)	12 (12.6)
Prosocial behaviors	8.32 ± 1.38	91 (95.8)	2 (2.1)	2 (2.1)

Internalizing and externalizing problems

When the SDQ domains were grouped more broadly, internalizing problems had a mean score of 4.62 ± 1.65 and were abnormal in 11.6% of siblings. Externalizing problems had a mean score of 4.21 ± 3.31 and were abnormal in 13.7% of siblings. These findings indicate that externalizing difficulties were slightly more common than internalizing difficulties in this sample.

The distribution of internalizing and externalizing problems is shown in Table 3.

**Table 3:** Distribution of parent-reported SDQ internalizing and externalizing composite scores among siblings of children with ADHD (N = 95). The dash (–) indicates that no borderline category was defined for the composite internalizing and externalizing scores. Internalizing score = emotional-symptoms score + peer-problems score; externalizing score = conduct-problems score + hyperactivity/inattention score. ADHD: attention‑deficit/hyperactivity disorder.

Domain	Mean ± SD	Normal, n (%)	Abnormal, n (%)
Internalizing score (range 0-20)	4.62 ± 1.65	84 (88.4)	11 (11.6)
Externalizing score (range 0-20)	4.21 ± 3.31	82 (86.3)	13 (13.7)

Most siblings had no parent-reported impact on the SDQ impact supplement; however, 14 siblings (14.7%) had an abnormal overall impact score. The distribution of impact scores is shown in Figure 3.

**Figure 3:**
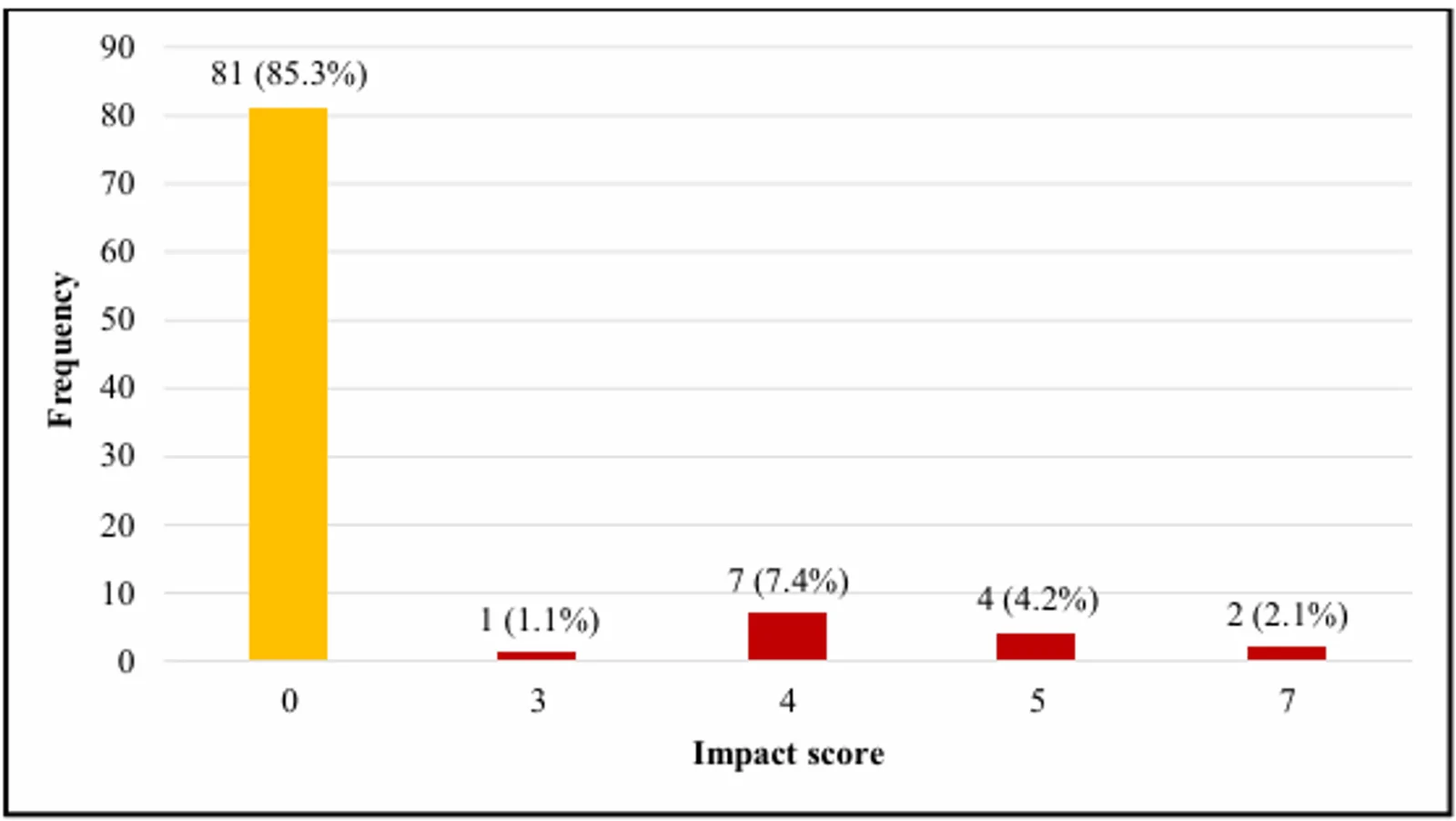
Distribution of impact scores on the Strengths and Difficulties Questionnaire (SDQ) among siblings of children with ADHD (N = 95). ADHD: attention‑deficit/hyperactivity disorder.

Associations of sociodemographic and family factors with SDQ scores

In exploratory bivariate analyses, borderline-or-abnormal emotional-symptom scores were associated with paternal education, maternal education, and maternal occupation. Borderline-or-abnormal conduct-problem scores were associated with male sex, a family history of psychiatric disorder, and parent-reported sibling involvement in caring for the child with ADHD. Borderline-or-abnormal hyperactivity/inattention scores were associated with male sex, paternal education, family history of psychiatric disorder, and parent-reported sibling involvement in caring for the child with ADHD. Borderline-or-abnormal peer-problem scores were associated with the number of siblings. These associations should be interpreted cautiously because several subgroup comparisons had sparse cell counts.

Higher proportions of borderline-or-abnormal conduct-problem and hyperactivity/inattention scores were observed among siblings categorized by parents as being occasionally involved in caring for the child with ADHD; this finding should be interpreted cautiously because the category sizes were small and caregiving frequency was parent-reported without predefined time thresholds.

Table 4 summarizes the statistically significant bivariate associations between sociodemographic and family characteristics and SDQ domains.

**Table 4:** Statistically significant bivariate associations between sociodemographic and family characteristics and borderline-or-abnormal SDQ domain scores among siblings of children with ADHD (N = 95). The dash (–) indicates not applicable or non‑reported values. Only variables with statistically significant bivariate associations (p<0.05) are presented. For bivariate analyses, borderline and abnormal SDQ categories were combined and compared with normal scores. Therefore, frequencies in this table differ from Table 2, which presents normal, borderline, and abnormal categories separately. Percentages based on small subgroup denominators, particularly maternal business occupation (n=1) and two siblings (n=3), should be interpreted cautiously. ADHD: attention‑deficit/hyperactivity disorder; SDQ: Strengths and Difficulties Questionnaire.

SDQ domain	Significant variable	Category with higher abnormal proportion	Borderline-or-abnormal score, n (%)	Comparison category	Borderline-or-abnormal score, n (%)	Chi-square	p-value
Emotional problems	Father's education	Undergraduate	4 (16.7)	Graduate	4 (5.6)	4.83	0.027
	Mother's occupation	Business	1 (100)	Housewife / Service	6 (7.4) / 1 (7.7)	10.99	0.004
	Mother's education	Undergraduate	5 (10.0)	Graduate	3 (6.7)	3.9	0.048
Conduct problems	Gender	Male	15 (21.7)	Female	2 (7.7)	4.53	0.033
	Family history of psychiatric disorder	Present	7 (31.8)	Absent	10 (13.7)	3.77	0.032
	Time spent caring for the child with ADHD	Occasionally	12 (85.7)	Almost Always / No Involvement	5 (6.3) / 0 (0)	51.45	<0.001
Hyperactivity problems	Gender	Male	10 (14.5)	Female	1 (3.8)	4.15	0.041
	Father's education	Undergraduate	4 (16.7)	Graduate	7 (9.9)	6.16	0.013
	Family history of psychiatric disorder	Present	5 (22.2)	Absent	6 (8.2)	3.47	0.036
	Parent-reported sibling involvement in caring for the child with ADHD	Occasionally	10 (71.4)	Almost always/no involvement	1 (1.3) / 0 (0)	57.45	<0.001
Peer relationship problems	Number of siblings	Two	3 (100)	One	32 (34.8)	5.31	0.047

Summary of significant findings

Across SDQ domains, statistically significant bivariate associations were observed with parental education, maternal occupation, sex, family history of psychiatric disorder, number of siblings, and parent-reported sibling involvement in caring for the child with ADHD. Associations involving sparse subgroup categories should be interpreted cautiously. Peer-problem scores were significantly associated only with the number of siblings; however, this result was based on three participants with two siblings and should be interpreted cautiously.

## Discussion

In this tertiary-care sample of siblings of children with ADHD, abnormal parent-reported SDQ scores were identified in a subset of participants, most commonly in peer-problem, conduct-problem, and hyperactivity/inattention domains. Because the study did not include a comparison group, these findings do not establish whether emotional and behavioral difficulties were more common than among children without an ADHD-affected sibling or in the general population. Rather, the findings describe the distribution of parent-reported SDQ scores within this clinical sample. Previous studies have reported psychosocial, behavioral, and peer-related difficulties among siblings of children with ADHD, although differences in settings, sampling methods, informants, measures, and comparison groups limit direct comparisons across studies [10-18].

Previous research, including a study from Thailand, has reported emotional and behavioral difficulties among siblings of children with ADHD [18]. However, differences in study design, recruitment setting, participant characteristics, informants, measures, and score classifications limit direct comparison with the present findings. The present study adds descriptive information from a tertiary psychiatric setting in Bangladesh, where sibling mental health in families affected by ADHD has received limited research attention.

The prominence of peer, conduct, and hyperactivity/inattention domains in this sample may reflect a combination of familial vulnerability and family or social circumstances. Previous literature suggests that siblings of children with ADHD may experience sibling conflict, disrupted routines, reduced privacy, perceived unequal parental attention, and caregiving demands, which may affect adjustment and family functioning [6-15]. However, these factors were not directly measured in the present study and should not be considered established explanations for the observed SDQ score patterns.

Parental education was associated with selected SDQ domains in exploratory bivariate analyses. Lower educational attainment may be related to differences in family resources, health literacy, parenting practices, or care-seeking; however, these potential pathways were not measured in the present study. Prior research has documented parenting stress and family-level challenges among families of children with ADHD, but the specific role of parental education in sibling emotional and behavioral outcomes requires further investigation [6,7]. These possible explanations should therefore be regarded as hypotheses for future research rather than conclusions from the observed associations.

Maternal occupation was associated with selected SDQ domains; however, this finding requires considerable caution. The occupational groups were highly imbalanced, and the business category included only one mother. Previous research has examined associations between maternal employment and child mental-health and socioemotional outcomes, but such associations may depend on family circumstances, employment conditions, caregiving arrangements, and other contextual factors [21,22]. In the present study, percentages and statistical estimates involving the business category are unstable and should not be interpreted as evidence of an occupational effect. Larger studies with more balanced occupational groups and direct measures of caregiving burden, work conditions, and family resources are needed.

Male siblings had higher proportions of borderline-or-abnormal conduct-problem and hyperactivity/inattention scores in bivariate analyses. This association should be interpreted cautiously because male participants predominated in the sample, potentially limiting the precision and generalizability of sex-specific estimates. A parent-reported family history of psychiatric disorder was also associated with conduct-problem and hyperactivity/inattention scores. This pattern is compatible with possible shared familial vulnerability, as ADHD and other psychiatric conditions may cluster within families; however, the cross-sectional design and reliance on parent report do not permit conclusions about underlying mechanisms [16,17].

Parent-reported sibling involvement in caring for the child with ADHD was associated with conduct-problem and hyperactivity/inattention scores. This variable was reported by parents using the categories “almost always,” “occasionally,” and “no involvement,” without predefined quantitative time thresholds. Therefore, the observed association may reflect unmeasured factors such as family stress, severity of the index child’s difficulties, parental availability, socioeconomic circumstances, or other confounding factors. Reverse causality is also possible, as siblings with behavioral difficulties may be less able or less likely to participate in caregiving. Accordingly, this association should not be interpreted as evidence that caregiving causes conduct or hyperactivity problems. Family stress, disrupted routines, conflict, unequal parental attention, and caregiving demands have been discussed as potential influences on sibling adjustment in families affected by ADHD, but these pathways were not directly assessed in this study [6-15].

An exploratory bivariate association was observed between borderline-or-abnormal peer-problem scores and the number of siblings. However, this finding was based on only three participants with two siblings, all of whom had borderline-or-abnormal peer-problem scores. The resulting 100% proportion is unstable and should be interpreted cautiously. Previous research recognizes that sibling relationships and family structure may influence children’s social and emotional development, but this study cannot determine the mechanism underlying the observed association [8,9,13,14].

Clinical implications

Clinicians may consider asking about sibling well-being during family assessment of children with ADHD. They should ask about siblings’ emotional and behavioral well-being, peer relationships, school functioning, and caregiving experiences; brief tools such as the Strengths and Difficulties Questionnaire may help identify siblings requiring further evaluation [20].

Family psychoeducation should address the impact of ADHD on the whole household. Parents may benefit from guidance on equitable attention, predictable routines, reducing sibling conflict, limiting inappropriate caregiving responsibilities, and encouraging positive sibling interactions [10-15].

Siblings with clinically significant difficulties may benefit from supportive counselling, family-based interventions, social-skills support, or referral to child and adolescent mental health services. Where peer or school difficulties are present, school-based support may be considered with parental consent.

Strengths and limitations

This study addresses an under-researched topic in Bangladesh and used a standardized screening instrument within a tertiary child psychiatry service. It also examined multiple sociodemographic and family-related factors among siblings of children with ADHD. However, the cross-sectional design precludes causal inference, and the absence of a comparison group prevents conclusions about whether the observed parent-reported SDQ score distribution differs from that among children without an ADHD-affected sibling or from the general population. The single-center purposive sample limits generalizability, and the modest sample size may have reduced power for some subgroup analyses. In addition, only bivariate associations were examined. Multivariable regression analysis was not performed because several SDQ outcome domains had a small number of abnormal cases, which would have resulted in unstable estimates and a high risk of overfitting. Therefore, residual confounding cannot be excluded. Several subgroup categories and SDQ outcome categories had small cell frequencies; therefore, the corresponding chi-square results should be interpreted cautiously. Several exposure categories contained very small numbers; therefore, estimates based on these subgroups, including 100% values, are unstable and should be interpreted cautiously. Furthermore, SDQ outcomes, family psychiatric history, and sibling involvement in caring for the child with ADHD were based on parent report and may be subject to recall, perception, or social-desirability bias. Detailed measures of parental stress, sibling relationship quality, family functioning, parenting practices, ADHD severity or comorbidity in the index child, and quantitative caregiving time were not collected; these unmeasured factors may have influenced the observed associations.

## Conclusions

Abnormal parent-reported SDQ scores were identified among a subset of siblings of children with ADHD attending a tertiary psychiatric service in Bangladesh, particularly in peer-problem, conduct-problem, and hyperactivity/inattention domains. Exploratory bivariate associations were observed with selected sociodemographic and family-related factors. These findings should be interpreted cautiously because of the cross-sectional design, absence of a comparison group, small and imbalanced subgroups, and lack of multivariable adjustment. Larger multicenter studies with comparison groups and longitudinal follow-up are needed to clarify the extent, temporal relationships, and potential determinants of these difficulties.
